# On the simulation and interpretation of substrate-water exchange experiments in photosynthetic water oxidation

**DOI:** 10.1007/s11120-024-01084-8

**Published:** 2024-03-21

**Authors:** Petko Chernev, A. Orkun Aydin, Johannes Messinger

**Affiliations:** Molecular Biomimetics, Department of Chemistry – Ångström Laboratory, 75120 Uppsala, Sweden

**Keywords:** Photosystem II, Oxygen-evolving complex, Mechanism of water oxidation, Membrane inlet mass spectrometry (MIMS), Substrate-water exchange

## Abstract

Water oxidation by photosystem II (PSII) sustains most life on Earth, but the molecular mechanism of this unique process remains controversial. The ongoing identification of the binding sites and modes of the two water-derived substrate oxygens (‘substrate waters’) in the various intermediates (S_*i*_ states, *i* = 0, 1, 2, 3, 4) that the water-splitting tetra-manganese calcium penta-oxygen (Mn_4_CaO_5_) cluster attains during the reaction cycle provides central information towards resolving the unique chemistry of biological water oxidation. Mass spectrometric measurements of single- and double-labeled dioxygen species after various incubation times of PSII with H_2_^18^O provide insight into the substrate binding modes and sites via determination of exchange rates. Such experiments have revealed that the two substrate waters exchange with different rates that vary independently with the S_*i*_ state and are hence referred to as the fast (W_f_) and the slow (W_S_) substrate waters. New insight for the molecular interpretation of these rates arises from our recent finding that in the S_2_ state, under special experimental conditions, two different rates of W_S_ exchange are observed that appear to correlate with the high spin and low spin conformations of the Mn_4_CaO_5_ cluster. Here, we reexamine and unite various proposed methods for extracting and assigning rate constants from this recent data set. The analysis results in a molecular model for substrate-water binding and exchange that reconciles the expected non-exchangeability of the central oxo bridge O5 when located between two Mn(IV) ions with the experimental and theoretical assignment of O5 as W_S_ in all S states. The analysis also excludes other published proposals for explaining the water exchange kinetics.

**Supplementary Information:**

The online version contains supplementary material available at 10.1007/s11120-024-01084-8.

## Introduction

**Fig. 1:**
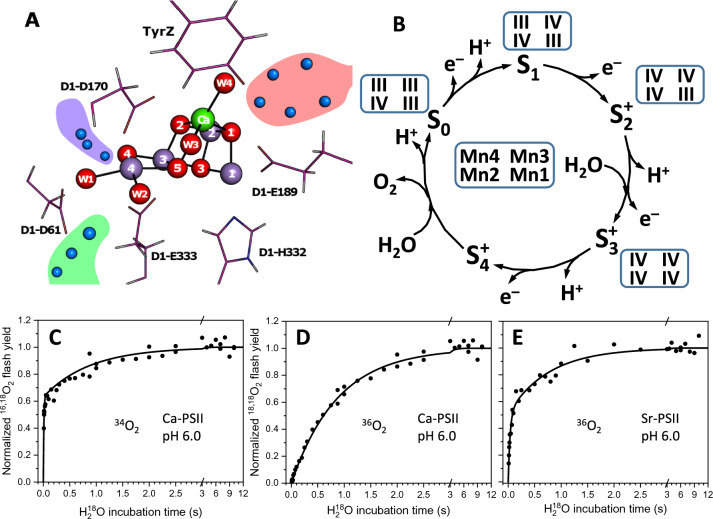
The oxygen-evolving complex of PSII, its reaction cycle, and substrate-water exchange experiments. **A** Structure of the Mn_4_CaO_5_ cluster and its surrounding (PDB: 7RF1). Manganese is shown in magenta, oxygen in red, calcium in green, water molecules around the active site in blue. Water-filled channels are represented with red (O1 channel), blue (O4 channel), and green (Cl1 channel) areas. **B** S state cycle of the Mn cluster. The formal oxidation states of the four Mn atoms in the four quasi-stable states are given in the boxes next to the state. **C**, **D** TR-MIMS measurements of the water exchange in the S_2_ state of Ca-PSII showing the single ^18^O-labeled (**C**) and double-labeled (**D**) O_2_ yield at pH 6. **E** double-labeled O_2_ yield of Sr-PSII. Points in **C**–**E** show individual experimental data points, and lines show exponential fits; redrawn from Ref. de Lichtenberg and Messinger (2020)

Photosystem II (PSII) catalyzes the oxidation of water into molecular oxygen and protons. The reaction happens at the Mn_4_CaO_5_ cluster (Fig. 1A) in the oxygen-evolving complex (OEC) of PSII, which is oxidized stepwise within a catalytic cycle that is driven by light-induced charge separations in the reaction center of PSII. Thus, the OEC is going through five intermediate states, S_0_ through S_4_, where the subscript indicates the number of stored oxidizing equivalents (Fig. 1B) (Kok et al. 1970). Molecular oxygen is released in the transition of the highly reactive S_4_ state to S_0_ in which also one substrate water binds, while the second substrate water is inserted into the cluster during the S_2_ → S_3_ transition (Dau et al. 2010; Pantazis 2018; Kern et al. 2018; Lubitz et al. 2019; Junge 2019; Suga et al. 2019; Ibrahim et al. 2020; Yamaguchi et al. 2022b; Shevela et al. 2023). High-resolution structures have been reported first for the dark-stable S_1_ state (Umena et al. 2011; Suga et al. 2015; Tanaka et al. 2017; Young et al. 2016), and recently also for the S_2_, S_3_, and S_0_ states (Kern et al. 2018; Suga et al. 2017) and several time points during the S_3_ → S_4_ → S_0_ transition (Bhowmick et al. 2023).

Despite the great advancement in the structural resolution of PSII in its reaction cycle, the mechanism of the O–O bond formation remains controversial (Vinyard et al. 2013, 2017; Li and Siegbahn 2015; Lubitz et al. 2019; Yamaguchi et al. 2022a; Greife et al. 2023; Bhowmick et al. 2023; Shevela et al. 2023). An important step towards resolving some of the open questions would be to identify the two substrate waters in all the S states. Presently, the only technique that can provide a unique signature for the substrate water molecules is time-resolved membrane inlet mass spectrometry (TR-MIMS) in combination with H_2_^16^O/H_2_^18^O exchange (Messinger et al. 1995; Messinger 2004; Hillier and Wydrzynski 2008; Cox and Messinger 2013). In this method, H_2_^18^O is rapidly injected into a PSII sample and the isotopic composition of the O_2_ produced by flash illumination after various incubation times is measured in order to determine the exchange rates of the two substrate waters.

The TR-MIMS measurements reveal a fast-exchanging (W_f_) and a slowly exchanging (W_S_) substrate water. The exchange of W_S_ can be resolved in all quasi-stable S states and usually happens on time scales from tens of milliseconds (S_0_ state) through seconds (S_2_ and S_3_) to tens of seconds (S_1_). The half-time of W_f_ exchange in the S_2_ and S_3_ states is also resolvable in the TR-MIMS measurements, at around 7 to 25 ms, while in the S_0_ and S_1_ states W_f_ exchanges faster than it can be resolved with this method (Hillier et al. 1998; Hillier and Wydrzynski 2000; Nilsson et al. 2014a; Cox and Messinger 2013). The biphasic behavior of the single ^18^O-labeled O_2_ yield (denoted ^34^Y) reflects the distinct exchange kinetics of the two substrate waters (Fig. 1C), while the yield of the double-labeled O_2_ (^36^Y) rises usually monophasic and is mostly determined by the exchange of W_s_ (Fig. 1D). Therefore, the TR-MIMS data can be analyzed by fitting a single-exponential curve to the ^36^Y-vs-time plot, while ^34^Y requires two exponential components (Messinger et al. 1995; Hillier and Wydrzynski 2000):

$$ {}^{34}{\text{Y}} = a\left( {1 - e^{{ - k_{f} t}} } \right) + \left( {1 - a} \right)\left( {1 - e^{{ - k_{s} t}} } \right) $$

1 $$ {}^{36}{\text{Y}} = 1 - e^{{ - k_{s} t}} $$

These equations describe a system with pseudo-first-order kinetics, where exchange happens at two independent sites with apparent rate constants *k*_f_ and *k*_s_, under the condition that *k*_f_ ≫ *k*_s_. The parameter *a* describes the exponential component proportions of the fast and slow exchange in the ^34^Y data, and it can be shown (Messinger et al. 1995) that *a* should be a function of the isotope contents of the bulk water in the sample:

2 $$a=\frac{{\alpha }_{{\text{f}}}\left(1-{\alpha }_{{\text{in}}}\right)+{\alpha }_{{\text{in}}}\left(1-{\alpha }_{{\text{f}}}\right)}{2{\alpha }_{{\text{f}}}\left(1-{\alpha }_{{\text{f}}}\right)},$$

where *α*_in_ and *α*_f_ are the initial and final H_2_^18^O enrichment correspondingly; *a* is approximately equal to 0.5/(1 − *α*_f_) if the initial enrichment (estimated to be around 0.7% in these experiments) is ignored.

**Fig. 2:**
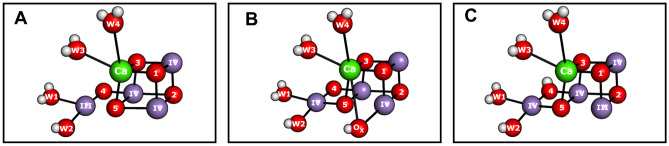
Proposals for high spin (*g* ≥ 4.1) conformation of the OEC in S_2_ state. **A** Closed cubane model in which the ‘dangling’ Mn4 is a pentacoordinate Mn(III) ion (Pantazis et al. 2012; Isobe et al. 2012; Bovi et al. 2013); **B** early water binding between Mn1 and Ca (Pushkar et al. 2019; de Lichtenberg and Messinger 2020) in which Mn2 or Mn3 maybe the only Mn(III) (Pushkar et al. 2019); **C** proton isomer of the open cubane conformation in which, compared to S_2_^LS^, a proton is moved from W1 to O4 (Corry and O’Malley 2019). Manganese atoms are shown in magenta, calcium in green, and oxygen in red with arabic numbers used as indices, whereas the roman numbers are showing the oxidation states of Mn ions

The S_2_ state can exist in at least two different conformations, one having an EPR signal centered at *g* = 2.0 (low spin, LS) and the other at 4.1 (high spin, HS) (Dismukes and Siderer 1981; Zimmermann and Rutherford 1984; Kim et al. 1992). Only the *g* = 2.0 conformation (Fig. 1A) is observed in untreated cyanobacterial PSII in serial crystallography experiments at room temperature (Kern et al. 2018; Li et al. 2021), but EPR experiments reveal that by Ca/Sr exchange and/or high pH a HS conformation can be induced that occurs at *g* = 4.8–4.9, indicating that it may differ in structure compared to S_2_^HS^ of plant PSII (Boussac et al. 2018). At least three different conformations have been suggested for the Mn_4_CaO_5/6_ cluster in the S_2_^HS^ state (Fig. 2), which all were shown by DFT calculations to give the S = 5/2 spin state (Pantazis et al. 2012; Corry and O’Malley 2019; Pushkar et al. 2019), while models B and C were also favorably compared to x-ray spectroscopy results of S_2_^HS^ (Pushkar et al. 2019; Chatterjee et al. 2019). It is noted that the three proposals are not mutually exclusive and that the ability of the Mn_4_CaO_5_ cluster to transiently attain additional conformations in the S_2_ state forms the basis of the proposal for exchanging the central O5 bridge (Siegbahn 2013).

The HS and LS conformations may exhibit different substrate-water exchange kinetics, and if a sample contains significant (> 10%) fractions of both conformations, then Eq. (1) may not be a suitable description of the system. In that case, an additional kinetic component may be observed in the ^34^Y and ^36^Y signals, and has been fitted by the following expressions (de Lichtenberg and Messinger 2020):

$$ {}^{34}{\text{Y}} = a\left( {1 - e^{{ - k_{f} t}} } \right) + \left( {1 - a} \right)\left[ {b\left( {1 - e^{{ - k_{i} t}} } \right) + \left( {1 - b} \right)\left( {1 - e^{{ - k_{s} t}} } \right)} \right] $$

3 $$ {}^{36}{\text{Y}} = b\left( {1 - e^{{ - k_{i} t}} } \right) + \left( {1 - b} \right)\left( {1 - e^{{ - k_{s} t}} } \right). $$

**Scheme 1:**
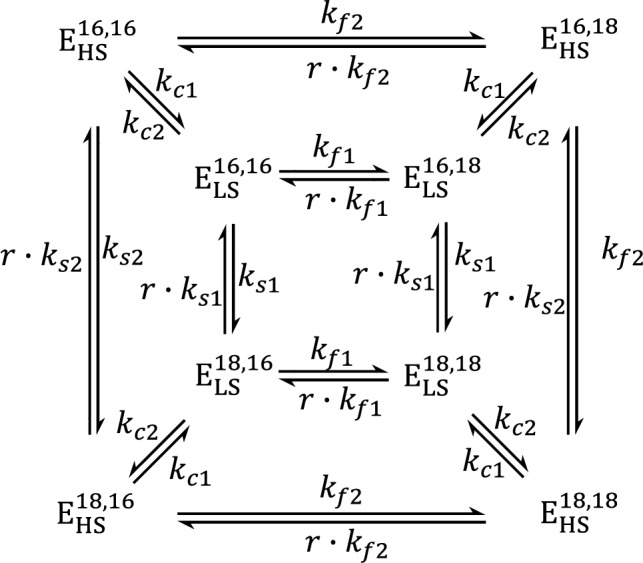
Substrate-water exchange reactions in a double-conformation model of PSII. Modified after (Huang and Brudvig 2021). E_HS_ and E_LS_ signify the conformations of the HS and LS states of the Mn_4_CaO_5_ cluster in PSII, while the superscripts (E^Ws,Wf^) indicate the oxygen isotope bound in the binding sites of the slowly (W_S_) and fast (W_f_) exchanging substrate waters. The exchange rates of W_f_ and W_S_ in E_LS_, E_HS_ are denoted *k*_f1_*, k*_f2_ and *k*_s1_*, k*_s2_, respectively, while the rates for the E_HS_/E_LS_ interconversion are noted as *k*_c1_ and *k*_c2_

The *k*_f_ rate constant, as before, reflects the fast-exchanging water in both conformations; since the data did not reveal two distinct phases for the fast water exchange, W_f_ was modeled as a single exchange component. For W_S_, however, two components were resolved, of which the faster, *k*_*i*_, is assigned to the exchange of W_s_ in HS conformation of the S_2_ state (*k*_s2_ in Scheme 1). For the slower component, *k*_s_, of W_s_ exchange two possible explanations were given: it could correspond to the W_S_ exchange in the LS conformation exhibiting the *g* = 2.0 EPR multiline signal (*k*_s1_ in Scheme 1), or to the rate constant of conformational change, *k*_c2_ (Scheme 1) of the LS state (E_LS_) to the HS state (E_HS_). The coefficient *b* depends on the equilibrium ratio of the two conformations.

Explicit kinetic modeling of the water exchange has also been done. A numerical solution of the differential equations describing a model with two binding sites and two conformations (Scheme 1) has been applied previously and resulted in exchange rates consistent with those derived by Eq. (3) (de Lichtenberg and Messinger 2020). Recently, Huang and Brudvig (2021) developed the analytical solution for this model, potentially allowing for a more accurate description of the water exchange measurements. However, due to the many terms in the complete analytical solution it is not possible to see the physical meaning and derive unique solutions. Thus, Huang and Brudvig examined some approximations to simplify the expressions. Consistent with our previous work, they conclude that the expressions can be reduced to the form shown in Eq. (3) under the following approximations: (a) the rate of conformational change is much slower than the rates of water exchange; (b) the rate of exchange of W_s_ is significantly slower than that of W_f_, so that W_f_ exchange is essentially complete before W_S_ exchange commences; (c) the fast-exchange rate constants in the two conformations are equal. Due to the first approximation, the rates of conformational change are not present in the exponential factors of the simplified equations, and thus the authors concluded, in contrast to de Lichtenberg and Messinger (2020), that the parameter *k*_s2_ in Eq. (3) cannot correspond to the rate of conformational change between the LS and HS isomers, but only to the rate constant of W_s_ exchange in the LS conformation. However, this specific approximation is not always satisfied, as seen even in some of the simulations presented in the paper of Huang and Brudvig (2021).

In the following, we revisit the analytic description developed by Huang and Brudvig (2021) and examine other possible simplifications and corrections.

## Results and discussion

### Non-zero initial H_2_^18^O enrichment

The initial conditions (concentrations of each state at time zero) of the system of differential equations are not discussed by Huang and Brudvig (2021). While the coefficients inside of the exponential functions do not depend on the initial conditions, the coefficients in front of the exponentials do. Examining the coefficients given in Ref. Huang and Brudvig (2021), it can be deduced that the authors assumed that at time zero all centers have H_2_^16^O, and that the equilibrium between the two conformations (dictated by the values of the conformation change rate constants) has already been established at time zero. The first assumption, about all centers holding ^16^O, is not exactly correct due to the natural abundance of ^18^O in normal water and due to leakage from the syringe used for injection of labeled water; it has been previously estimated that around 0.7% of the water at time zero has ^18^O (de Lichtenberg and Messinger 2020). This value is relatively small and does not affect the kinetics of the exchange; however, it may affect the simulations and fitting of the exchange data at early times. Including this value in the model is straightforward, and the equations describing the yield of single- and double-labeled oxygen for the model with two exchange sites and two conformations are now given by:

$$ {}^{34}{\text{Y}} \propto \frac{2r}{{\left( {1 + r} \right)^{2} }}\left[ { - \frac{1}{r}q^{2} C_{1} \left( t \right) - \frac{r - 1}{{2r}}qC_{2} \left( t \right) - \frac{r - 1}{{2r}}qC_{3} \left( t \right) + 1} \right] $$

$$ {}^{36}{\text{Y}} \propto \frac{1}{{\left( {1 + r} \right)^{2} }}\left[ {q^{2} C_{1} \left( t \right) - qC_{2} \left( t \right) - qC_{3} \left( t \right) + 1} \right] $$

4 $${C}_{i}(t)={c}_{i}^{+}{e}^{{\lambda }_{i}^{+}t}+{c}_{i}^{-}{e}^{{\lambda }_{i}^{-}t}.$$

The equations are identical with the ones in Ref. Huang and Brudvig (2021) (also shown in the Supporting Information), with the addition of the term *q* that describes the dependence on the initial H_2_^18^O enrichment:

5 $$q=1-\frac{1+r}{1+{r}_{0}}=1-\frac{{\alpha }_{{\text{in}}}}{{\alpha }_{{\text{f}}}}.$$

Here, *r* is the equilibrium ratio $\frac{\left[{{\text{H}}}_{2}^{16}{\text{O}}\right]}{\left[{{\text{H}}}_{2}^{18}{\text{O}}\right]}$ after the H_2_^18^O injection, and *r*_0_ is this ratio before the injection. The pre-exponential coefficients *c* and the exponential coefficients *λ* have the same expressions as given in Huang and Brudvig (2021) and in the Supporting Information. The inclusion of this correction to the simulations results in a change that is similar to a shift of the simulated curves to earlier times (see SI Fig. S1a).

### HS/LS equilibrium during the substrate exchange

The second assumption that the conformation equilibrium between S_2_^LS^ and S_2_^HS^ is already reached at time zero of H_2_^18^O incubation is presumably correct for short incubation times when the H_2_^18^O injection comes long (10–20 s) after the first flash that advances S_1_->S_2_, allowing enough time for the conformation equilibration to occur before the injection of labeled water. By contrast, for long incubation times, the injection happens soon after the first flash that generates S_2_. Here, the situation is less clear, since low temperature illumination (200 K) of PSII samples in the S_1_ state leads predominantly to the formation of S_2_^LS^, which only upon short warming transforms under suitable conditions into S_2_^HS^ (Boussac et al. 2018). The rate of this process is presently not well characterized. By contrast, others have concluded on the basis of DFT calculations that the ratio of S_2_^HS^ to S_2_^LS^ depends on the distribution of two conformations of the S_1_ state (Drosou et al. 2021). Even if that were the case, the equilibrium concentration between the respective conformations would be likely S state dependent and require a presently unknown time to establish. Due to these uncertainties, and the complexity of incorporating this additional equilibration that occurs over variable times into an already highly sophisticated model, we accept the assumption and make no attempt to include it here.

### Non-instant injection

In reality, the injection of H_2_^18^O into the PSII suspension and the subsequent mixing is not instant. The injection and mixing can be observed using a fluorescent dye (PSII or fluorescein) (Messinger et al. 1995; Nilsson et al. 2014a), and is approximately linear, taking place within around 6 ms. To evaluate the effect of the mixing on the exchange curves, we consider the exchange at a single site. The relative fraction of sites with labeled water, *E*^18^*,* can be described by:

6 $$\frac{{\text{d}}{E}^{18}}{{\text{d}}t}=k\left(\mathrm{\alpha }(t)-{E}^{18}(t)\right),$$

where *k* is the apparent rate constant of the exchange, and *α* is the enrichment of labeled water in the bulk. If *α* is a constant equal to the final enrichment *α*_f_, this has the solution:

7 $${E}_{\mathrm{instant mixing}}^{18}\left(t\right)={\alpha }_{{\text{in}}}+({\alpha }_{{\text{f}}}-{\alpha }_{{\text{in}}})\left(1-{e}^{-kt}\right).$$

This would describe the situation where the injection of labeled water is instantaneous and happens at *t* = 0.

Due to the time course for the injection and mixing, the enrichment is initially not constant but increases linearly, starting at *t* = 0 at *α*_in_ until a time *t* = *t*_*m*_ (which is approximately 6 ms for our experiments), when it reaches *α*_f_:

8 $$\alpha \left(t\right)=\left[\begin{array}{c}{\alpha }_{{\text{in}}}+\frac{{\alpha }_{{\text{f}}}-{\alpha }_{{\text{in}}}}{{t}_{m}}t\, {\text{if}} \,0\le t\le {t}_{m}\\ {\alpha }_{{\text{f}}}\,{\text{if}} \,t\ge {t}_{m}\end{array}\right.$$

In the two regions, the solution of (6) is now:

9 $${E}_{{\text{linear}} {\text{mixing}}}^{18}\left(t\right)=\left[\begin{array}{c}{\alpha }_{{\text{in}}}+\left({\alpha }_{{\text{f}}}-{\alpha }_{{\text{in}}}\right)\frac{\left({e}^{-kt}-1+kt\right)}{k{t}_{m}}\, {\text{if}} \,0\le t\le {t}_{m}\\ {\alpha }_{{\text{in}}}+\left({\alpha }_{{\text{f}}}-{\alpha }_{{\text{in}}}\right)\left(1-{e}^{-k\left(t-{t}_{k}\right)}\right)\, {\text{if}} \,t\ge {t}_{m}\end{array}\right.$$

Since all points of the water exchange data are measured at times *t* ≥ *t*_*m*_ = 6 ms, only the second time region in Eq. (9) is relevant. In this region, the expression for non-instantaneous injection (9) differs from the one for instantaneous injection (7) only by the factor *t*_*k*_, in a way that one is a time-shifted version of the other, with a time shift given by *t*_*k*_:

10 $${E}_{{\text{linear}} {\text{mixing}}}^{18}\left(t\right)={E}_{{\text{instant}} {\text{mixing}}}^{18}\left(t-{t}_{k}\right).$$

The coefficient *t*_*k*_ depends on the apparent rate constant of water exchange *k*:

11 $${t}_{k}=\frac{{\text{ln}}\left(\frac{{e}^{k{t}_{m}}-1}{k{t}_{m}}\right)}{k}.$$

The value of *t*_*k*_ approaches *t*_*m*_/2 = 3 ms for small *k*, staying close to that value for most of the relevant values of *k*, only increasing above 3.3 ms for *k* > 200 s^−1^ (see SI Fig. S2 for a plot of *t*_*k*_ as a function of *k*). At higher values of *k*, the exchange happens faster than the time resolution of the TR-MIMS experiment, with or without the linear mixing correction. Therefore, we simply use a value of *t*_*k*_ = 3 ms and expression (10) to approximate the effect of non-instant injection and mixing. The inclusion of this correction to the simulations results in a shift of the simulated curves to later times (see SI Fig. S1b), which is in opposite direction of the correction for non-zero initial H_2_^18^O enrichment.

### Water exchange in the S_3_ state affecting the S_2_ state exchange measurements

In the TR-MIMS experiments, when water exchange in the S_2_ state is measured, the PSII sample is first brought into the S_2_ state by one excitation light flash, then labeled water is injected into the sample, and after a varying incubation time two more light flashes are given to drive the O_2_ evolution reaction. The first of the two extra flashes brings the sample to the S_3_ state, where it spends a very short time, usually 10 ms, before the next flash that drives the S_3_ → S_0_ + O_2_ step. The time is short, in order to minimize the effect on the observed isotope ratios due to exchange in the S_3_ state, but must remain long enough to allow re-opening of the acceptor side and, thereby, advancement of a significant fraction to S_0_ coupled to O_2_ production. We note that for short H_2_^18^O incubation times in the S_2_ state, and for relatively fast S_3_ exchange kinetics, the effect might be significant and we thus recently started to account for it [see SI of de Lichtenberg et al. (2021)]. Knowing the water exchange rates in the S_3_ state, it is possible to account for the 10 ms S_3_ state exchange on the simulated S_2_ state exchange kinetics by doing an extra simulation for each time point, where one takes the final concentrations of the components from the S_2_ exchange simulation and uses them as initial concentrations for a 10 ms exchange using the S_3_ exchange rate constants [see SI of de Lichtenberg et al. (2021)]. This assumes that the fast- and slow-exchanging sites observed in S_2_ correspond to the fast- and slow-exchanging sites in S_3_. This correction results in the apparent shift of the simulated curves to earlier times (see SI Fig. S1c), opposite to the effect of the correction for non-instant mixing. This extra exchange in the S_3_ state is ignored in the model of Huang and Brudvig (2021).

Applying all three corrections to the simulation has their effects mostly canceling each other (see SI Fig. S1d), and thus not considering them should not affect the interpretation of the data in a major way, at least in most cases when the exchange in S_3_ is relatively slow. Nevertheless, in the rest of this work, all simulations are performed applying all three corrections.

### Interpretation of the water exchange data with the analytical solution of the two-site double-conformation model

The analytical solution of the two-site double-conformation model (Scheme 1) is a linear combination of a total of 8 exponential terms (Eq. 4) (Huang and Brudvig 2021). The arguments of the exponential functions are the H_2_^18^O incubation time multiplied by the eigenvalues of the rate constants matrix (one of them is zero, yielding a constant term). To make physical sense of the results, Huang and Brudvig applied two approximations that simplify the expressions: firstly, the rates of conversion between the two conformations (with rate constants ${k}_{{\text{c}}1}$ and ${k}_{{\text{c}}2}$) are both significantly slower than all other rates, and secondly, the fast exchange (with apparent rate constants ${k}_{{\text{f}}1}^{*}$ and ${k}_{{\text{f}}2}^{*}$) is significantly faster than the slow exchange (${k}_{{\text{s}}1}^{*}$ and ${k}_{{\text{s}}2}^{*}$). The authors then arrive at simple approximate expressions that show that the yield of double-labeled O_2_ (^36^Y) is a sum of two exponentials with rate constants equal to the two slow exchange constants, ${k}_{{\text{s}}1}^{*}$ and ${k}_{{\text{s}}2}^{*}$. Both the constants come from the third eigenvalue pair, ${\lambda }_{3}^{+/-}$ in Eq. (4) [Eq. 30 in Huang and Brudvig (2021)]; due to the assumption that ${k}_{f}^{*}\gg {k}_{s}^{*}$, the terms containing ${\lambda }_{1}^{+/-}$ and ${\lambda }_{2}^{+/-}$ cancel each other. The yield of single-labeled O_2_ (^34^Y) adds two more exponentials with constants equal to the fast-exchange constants. The rate constants of interconversion between the two conformations appear only in the pre-exponential coefficients, but not as parameters of the exponential functions in this simplification. However, while the approximation that ${k}_{f}^{*}\gg {k}_{s}^{*}$ is presumably valid under most circumstances, the assumption that ${k}_{s}^{*}{\gg k}_{c}$ may not hold in all cases; for example, in the simulations of the Sr^2+^-PSII data at pH 8.3 presented in Table 2 of Ref. Huang and Brudvig (2021) the conformation change rate constants ${k}_{{\text{c}}}$ are significantly larger than one of the rate constant of the slow exchange ${k}_{{\text{s}}}^{*}$.

A different simplification of the equations and a different interpretation of the experimental data is possible, which is not discussed by Huang and Brudvig (2021). In this approximation, only one of the slow exchange rates (${k}_{{\text{s}}1}^{*}$ or ${k}_{{\text{s}}2}^{*}$) needs to be significantly faster than only one of the rate constants of conformation change (${k}_{{\text{c}}1}$ or ${k}_{{\text{c}}2}$). This would lead to the other conformation change rate appearing in the approximate expressions for ^34^Y and ^36^Y. We first note that the (exact) expression for the third pair of eigenvalues can be rewritten as:

12 $${\lambda }_{3}^{+,-}=\frac{1}{2}\left[-\left({k}_{{\text{s}}1}^{*}+{k}_{{\text{s}}2}^{*}+{k}_{{\text{c}}1}+{k}_{{\text{c}}2}\right)\pm \sqrt{{\left({-k}_{{\text{s}}1}^{*}+{k}_{{\text{s}}2}^{*}+{k}_{{\text{c}}1}-{k}_{{\text{c}}2}\right)}^{2}+4{k}_{{\text{c}}1}{k}_{{\text{c}}2}}\right].$$

With the relaxed approximation requirements, we can neglect only the term $4{k}_{{\text{c}}1}{k}_{{\text{c}}2}$ in the above expression, which allows us to cancel the square and square root, yielding the following two approximate expressions for the eigenvalues:

13 $$\begin{array}{cc}{\lambda }_{3}^{+}\approx -\left({k}_{{\text{s}}1}^{*}+{k}_{{\text{c}}2}\right),& {\lambda }_{3}^{-}\approx -\left({k}_{{\text{s}}2}^{*}+{k}_{{\text{c}}1}\right)\end{array}.$$

If both ${k}_{{\text{s}}1}^{*}$ and ${k}_{{\text{s}}2}^{*}$ are significantly larger than ${k}_{{\text{c}}1}$ and ${k}_{{\text{c}}2}$ we still get, as in Huang and Brudvig (2021):

14 $$\begin{array}{cc}{\lambda }_{3}^{+}\approx -{k}_{{\text{s}}1}^{*},& {\lambda }_{3}^{-}\approx -{k}_{{\text{s}}2}^{*}\end{array}.$$

However, in the case when e.g., ${k}_{{\text{s}}1}^{*}$ ≪ ${k}_{{\text{c}}2}$, we get instead:

15 $$\begin{array}{cc}{\lambda }_{3}^{+}\approx -{k}_{{\text{c}}2},& {\lambda }_{3}^{-}\approx -{k}_{{\text{s}}2}^{*}\end{array}.$$

This would mean that the two phases observed in the yield of double-labeled O_2_ (^36^Y) would not reflect the exchange of W_S_ in the two conformations, but instead the rate of exchange in E_HS_ and the conversion of E_LS_ to E_HS_ (Scheme 1). This corresponds to the second interpretation obtained by de Lichtenberg and Messinger (2020) employing Eq. (3).

**Fig. 3:**
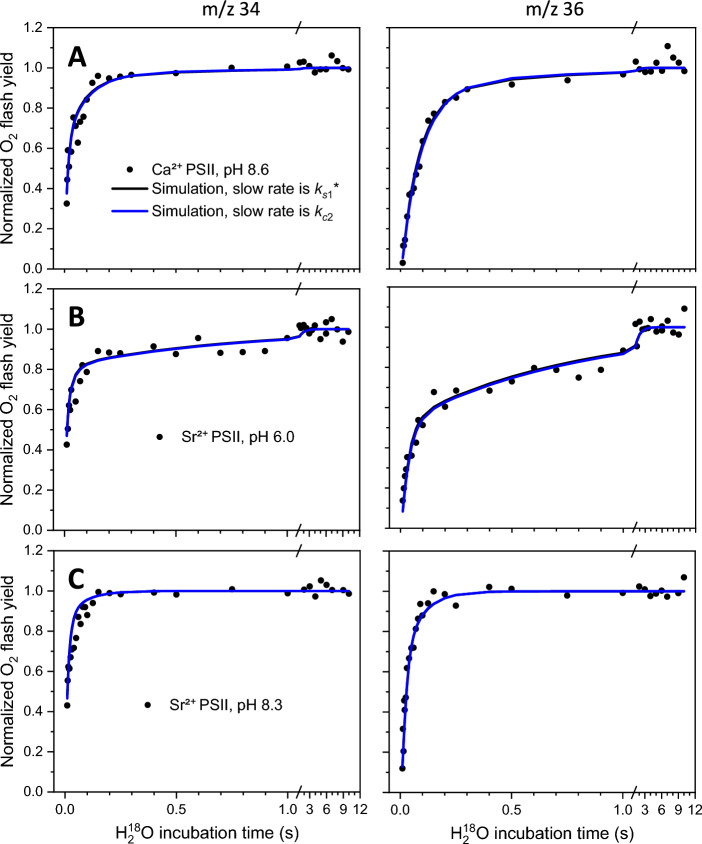
Substrate-water exchange data in two-conformation systems in the S_2_ state taken from Ref. de Lichtenberg and Messinger (2020) and simulated using our extended form of the double-conformation model (Scheme 1) presented in Huang and Brudvig (2021). The single-labeled O_2_ yield (^34^Y) is shown on the left, and the double-labeled O_2_ yield (^36^Y) is shown on the right. Black dots show individual experimental data points. The black and the blue curves (mostly overlapping) show simulations using rate constants given in Table 1, for the cases when the slowest kinetic component corresponds either to the slow water exchange in E_LS_ (${k}_{{\text{s}}1}^{*}$, black) or to the rate constant for the conversion of E_LS_ to E_HS_ (${k}_{{\text{c}}2}$, blue). The intermediate kinetic component is explained in all simulations by the slow water exchange in E_HS_. Corrections for initial enrichment (${\alpha }_{{\text{in}}}$ = 0.7%), non-instant injection (*t*_*k*_ = 3 ms), and exchange in S_3_ (with additional 10 ms exchange using rate constants ${k}_{{\text{f}}}^{*}=19.5,{k}_{{\text{s}}}^{*}=0.25$ s^−1^) are applied for all simulations. **A** Ca^2+^-PSII, pH 8.6; **B** Sr^2+^-PSII, pH 6.0; **C** Sr^2+^-PSII, pH 8.3

For example, using the values from the first simulation for the Sr-PSII sample at pH 8.3 presented in Ref. Huang and Brudvig (2021) and shown in Table 1, the exact values of the two eigenvalues (Eq. 12, not approximated) are ${\lambda }_{3}^{+}$ = − 11.96 s^−1^ and ${\lambda }_{3}^{-}$ = − 54.64 s^−1^. It is only these two kinetic phases that can be seen in the ^36^Y trace, although they are not clearly visibly separated, as the two values are not sufficiently different (Fig. 3C, blue trace). The ${\lambda }_{3}^{-}$ = − 54.64 s^−1^ component is associated with the slow water exchange in E_HS_, ${k}_{{\text{s}}2}^{*}$ = 50 s^−1^. The ${\lambda }_{3}^{+}$ = − 11.96 s^−1^ component, however, matches the approximations given (Eq. 15) very well (${\lambda }_{3}^{+}$ = − 11.96 s^−1^ ≈ − ${k}_{{\text{c}}2}$ = − 12 s^−1^) and does not match the approximations (Eq. 14) given in Ref. Huang and Brudvig (2021) (− 11.96 s^−1^ ≠ − ${k}_{s1}^{*}$ = − 1 s^−1^), and thus reflects the conversion of E_LS_ to E_HS_ and not the exchange in E_LS_.

**Table 1:** Summary of the rate constants obtained from the substrate-water exchange experiments (de Lichtenberg and Messinger 2020) using the analytical solution of the extended form (see above) of the double-conformation model presented by Huang and Brudvig (2021)

Sample	Model	${k}_{{\text{f}}1}^{*}$	${k}_{{\text{f}}2}^{*}$	${k}_{{\text{s}}1}^{*}$	${k}_{{\text{s}}2}^{*}$	${k}_{{\text{c}}1}$	${k}_{{\text{c}}2}$	${k}_{{\text{c}}2}$/${k}_{{\text{c}}1}$	Chi^2^
Ca^2+^-PSII, pH 8.6	Ref. Huang and Brudvig (2021)	94 (fixed)	73 ± 14	1.1 (fixed)	11.7 ± 1.3	0.084 ± 0.13	0.62 ± 0.63	7.5 ± 1.5	0.11
	This work	94 (fixed)	73 ± 20	0.01 (fixed)	11 ± 1	0.13 ± 0.1	1.5 ± 0.7	12 ± 4	0.11
Sr^2+^-PSII, pH 6.0	Ref. Huang and Brudvig (2021)	120 (fixed)	75 ± 21	1.0 (fixed)	28.6 ± 13.5	0.30 ± 0.30	0.32 ± 0.23	1.0 ± 0.2	0.14
	This work	120 (fixed)	75 (fixed)	0.01 (fixed)	26 ± 7	1.1 ± 0.4	1.3 ± 0.2	1.2 ± 0.2	0.14
Sr^2+^-PSII, pH 8.3	Ref. Huang and Brudvig (2021)	120 (fixed)	65 ± 9	1.0 (fixed)	50 ± 10	3.6 ± 2.9	12 ± 6	3.4 ± 1.8	0.18
	This work	120 (fixed)	65 (fixed)	11 ± 3	57 ± 14	0.6 ± 0.3	1.0 (fixed)	1.6 ± 1.5	0.18

${k}^{*}$ denotes apparent rate constants. The first subscript of ${k}^{*}$ denotes the kind of rate constant (fast or slowly exchanging), and ${k}_{{\text{c}}}$ is the conformational change rate constant. The second subscript of $k$ denotes the conformation, see Scheme 1. All rate constants are in unit s^−1^. The errors of the fit parameters are estimated from the bootstrapping distributions (shown in SI Figs. S4–S6 for the models in this work). The simulated curves are shown in Fig. 3

In other simulations, the kinetic components observed in ^36^Y do correspond to the slow water exchange in the two conformations, ${k}_{{\text{s}}1}^{*}$ and ${k}_{{\text{s}}2}^{*}$, see the Ca^2+^-PSII, pH 8.6 and Sr^2+^-PSII, pH 6.0 simulations taken from Ref. Huang and Brudvig (2021) in Table 1 and Fig. 3A and B, black traces (mostly covered). However, these are not the only possible simulations that give the same fit quality. Depending on the values at which we choose to fix ${k}_{{\text{s}}1}^{*}$, we can obtain simulations in which the simulated ^34^Y and ^36^Y curves are practically identical, but for which the slowest observed kinetic component (with a rate of around 1 s^−1^ for both samples) corresponds not to ${k}_{{\text{s}}1}^{*}$ but to ${k}_{{\text{c}}2}$ (blue traces in Fig. 3A and B). The two kinetic components in ^36^Y are especially well separated in the Sr^2+^-PSII, pH 6.0 simulations (Figs. 1B, S3), and correspond to the eigenvalues ${\lambda }_{3}^{+}$ = − 1.31 s^−1^ ≈ − ${k}_{{\text{c}}2}$ and ${\lambda }_{3}^{-}$ = − 27 s^−1^ ≈ − ${k}_{{\text{s}}2}^{*}$ for the blue trace in Fig. 3B. Two interpretations of the exchange data in the Sr^2+^-PSII sample at pH 8.3 are also possible, see for example the last simulation in Table 1 and the black traces in Fig. 3C, where the two kinetic components in ^36^Y correspond to ${k}_{{\text{s}}1}^{*}$ and ${k}_{{\text{s}}2}^{*}$. We, therefore, conclude, in line with our previous work (de Lichtenberg and Messinger 2020), that it is not possible to tell from the simulations if the slowest observed kinetic component in the TR-MIMS traces corresponds to the slow water exchange in the LS state or to the rate of conversion of E_LS_ to E_HS_.

## Discussion

The present re-evaluation of the analytical solution obtained by Huang and Brudvig (2021) for water exchange in the high spin (E_HS_) and low spin (E_LS_) conformations of the S_2_ state of photosystem II shows that the simplifications required for interpreting the results lead to the same two possibilities as proposed previously by de Lichtenberg and Messinger (2020): firstly (solution 1), the two kinetic phases in the ^36^Y data correspond to the slow exchange constants (*k*_s1_ and *k*_s2_) in the two conformations; this requires that the conformational equilibrium is much slower than the slow exchange rates in the two conformations. Secondly (solution 2), the faster of the slow exchange rates corresponds to the slow exchange in the S_2_^HS^ state (E_HS_) that promotes W_s_ exchange, while the slower ^36^Y rise reflects the rate of conversion of E_LS_ to E_HS_ that promotes W_s_ exchange [see also de Lichtenberg and Messinger (2020)]. A molecular interpretation of this important result with regard to the binding site of W_s_ in the Mn_4_CaO_5_ cluster is complicated by the fact that the structure of E_HS_ remains controversial due to the absence of reliable x-ray diffraction or cryoEM data of PSII in the S_2_^HS^ state(s) (Fig. 2A–C).

Previous substrate-water exchange measurements, advanced EPR experiments with ^17^O labeling, and computational studies have indicated O5 as the slow-exchanging substrate (Messinger 2004; Siegbahn 2009, 2013; Rapatskiy et al. 2012; Cox and Messinger 2013). In the well-established low spin (multiline) conformation of the S_2_ state (E_LS_ in Scheme 1), O5 is bound as a µ_3_-oxo bridge between Mn4, Mn3, and Ca, with both Mn ions being in the formal oxidation state IV (Fig. 1). Thus, O5 is expected to be rather exchange inert in the E_LS_ conformation of the S_2_ state (Hillier and Wydrzynski 2001, 2008; Tagore et al. 2006, 2007), consistent with solution 2 in which one of the states is exchange inert and the slower phase of the ^36^Y rise is reflecting the rate of E_LS_ to E_HS_ conversion. Siegbahn calculated an exchange mechanism for O5 in the S_2_^LS^ state (E_LS_ in Scheme 1) that involves water binding to Mn1 and proton transfer to O5 coupled with stepwise electron transfer from Mn1 via Mn3 to Mn4 (Siegbahn 2013). In this sequence, O5 finally ends up being fully protonated and ligated as terminal ligand at Mn4(III), where it then can exchange with water surrounding the cluster (‘cavity’ water). The calculated rate-limiting barrier for this transition is surprisingly close in energy to the experimentally determined value (Hillier and Wydrzynski 2000; Siegbahn 2013). However, it may also be possible that a modified solution 1 is at play in which the two rate constants ${k}_{{\text{s}}1}^{*}$ and ${k}_{{\text{s}}2}^{*}$ reflect the exchange in E_LS_ and E_HS_, respectively, and where the exchange rates are limited by the different activation barriers for water binding to Mn1, thus a structural change not explicitly included in Scheme 1 [see also discussion in de Lichtenberg and Messinger (2020)].

The second conformation (E_HS_ in Scheme 1), in which O5 exchanges faster, was associated with the high spin state on the basis of its prominence at high pH and its sensitivity to ammonia addition as well as Ca/Sr exchange (de Lichtenberg and Messinger 2020), properties of the high spin state established earlier by Boussac and coworkers (Boussac et al. 2018). Nevertheless, while the correlation appears convincing, it cannot be fully excluded that the state promoting W_s_ exchange is not identical to the high spin state.

Of the three dominant proposals for the high spin S_2_ state (S_2_^HS^), one involves a closed cubane conformation Fig. 2A) (Pantazis et al. 2012; Isobe et al. 2012; Bovi et al. 2013). In this conformation, O5 is bound as µ_3_-oxo bridge between Mn1, Mn3, and Ca, and both ligating Mn ions are in oxidation state IV. Thus, it is not obvious as to why O5 would exchange more rapidly in this conformation. By contrast, such a structure would be more likely to promote a faster W2 or O4 exchange, which are alternative assignments for W_s_ in the literature [for discussion, see de Lichtenberg and Messinger (2020), Huang and Brudvig (2021)]. However, while several theoretical studies consistently identify the high spin state with a closed cubane structure (Pantazis et al. 2012; Isobe et al. 2012; Bovi et al. 2013), several experimental approaches targeted to elucidating the high spin structure found no evidence for this conformation (Chatterjee et al. 2019; Pushkar et al. 2019).

In the second proposed structure of the S_2_^HS^ conformation (Fig. 2B), early water binding to the Mn1 site is suggested, mostly for alkaline conditions (Pushkar et al. 2019; de Lichtenberg and Messinger 2020). Such a state is similar in structure to one of the intermediates of Siegbahn’s proposed O5 exchange pathway (Siegbahn 2013; de Lichtenberg and Messinger 2020). Thus, it may be plausible that in this state O5 would exchange more rapidly, assuming that reaching the water(hydroxide)-bound state would be rate limiting in E_LS_ at neutral pH [see discussion in de Lichtenberg and Messinger (2020)].

In the third suggested S_2_^HS^ state structure (Fig. 2C), mostly proposed for neutral or slightly acidic conditions, a proton from W1 is shifted to the O4-bidge (Corry and O’Malley 2019). This proton shift would likely promote O5 exchange by either (i) making deprotonation of water to hydroxide easier during binding to Mn1 via providing a suitable base (W1 = OH^−^) and/or (ii) enabling O5 protonation via the trans effect of OH^−^ in the W1 position. Thus, also this structural proposal is consistent with the S_2_^HS^ (E_HS_) state being faster exchanging than the S_2_^LS^ (E_LS_) state.

Vinyard et al. (2015) and Vinyard and Brudvig (2017) proposed an exchange model for substrate water in which they aimed to reconcile their expectation that O5 is not exchangeable in the S_2_ and S_3_ states with experimental evidence that O5 can exchange in the S_1_ state with rates consistent with those of W_s_ (Rapatskiy et al. 2012). In their proposal, W2 bound to Mn4(III) is, in the S_0_ and S_1_ states, in exchange equilibrium with both the bulk water and O5; thus, it exchanges at an unresolved fast rate (together with W3) with bulk water, and additionally with a slow rate with O5. In the S_2_ and S_3_ states, the slow exchange of W2 with O5 is proposed to be blocked because O5 then binds between two Mn(IV) ions. Instead, it is proposed that the accumulation of a positive charge during the S_1_ → S_2_ transition dramatically slows the exchange of the Ca-bound W3. In this explanation, W_s_ binding would be consistent with the acceleration of its exchange upon Ca/Sr substitution, but in conflict with known exchange rates for Ca-bound water (Helm and Merbach 2005; Hillier and Wydrzynski 2008). On that basis, Vinyard and Brudvig propose that O–O bond formation occurs between W2 and W3 via nucleophilic attack. While interesting, this proposal is inconsistent with available substrate-water exchange data and kinetic considerations: since the two proposed substrates W2 and W3 exchange rapidly with the essentially endless pool of bulk water in the S_1_ and S_2_ states, and W2 equilibration with O5 is more than 100-fold slower than the unresolved rapid exchange with bulk water, no slow phase but instead an essentially instantaneous complete exchange of ‘W_s_’ (W2) would be observable in both the ^34^Y and ^36^Y data for the S_0_ and S_1_ states, in stark contrast to experimental observations that clearly reveal the slow exchange phase.

Huang and Brudvig (2021) concluded, on the basis of their analysis and by favoring the closed cube model for S_2_^HS^ (E_HS_), that W1 and/or W2 may be substrate waters. In contrast the team’s earlier publications (Vinyard et al. 2015; Vinyard and Brudvig 2017), they excluded that Ca-bound W3 and W4 can be a substrate due to their weak association with Ca. However, no specific suggestion regarding a mechanism was made.

In 2013, Vinyard and Dismukes published a molecular explanation for the substrate exchange rates (Vinyard et al. 2013). While several features are similar to our earlier proposal (Messinger 2004), such as employing a closed cube conformation and assigning W_f_ to W2 and W_s_ to O5, this interpretation is based on the low-oxidation state paradigm in which the S_1_ state has the oxidation states Mn_4_(III,III,III,III). Additionally, special emphasis was given to explain the S_*i*_ state dependence of the exchange rates by changes in the Jahn–Teller axes of the Mn(III) ions connected to O5. The main argument against this mechanism is the convincing evidence for the high-oxidation state paradigm (Yachandra et al. 1996; Haumann et al. 2005; Kulik et al. 2007; Siegbahn 2009; Cox et al. 2014; Krewald et al. 2015; Cheah et al. 2020) and the experimental verification that the S_3_ state contains an extra oxygen bridge (Suga et al. 2017; Kern et al. 2018), see however Wang et al. (2021).

**Fig. 4:**
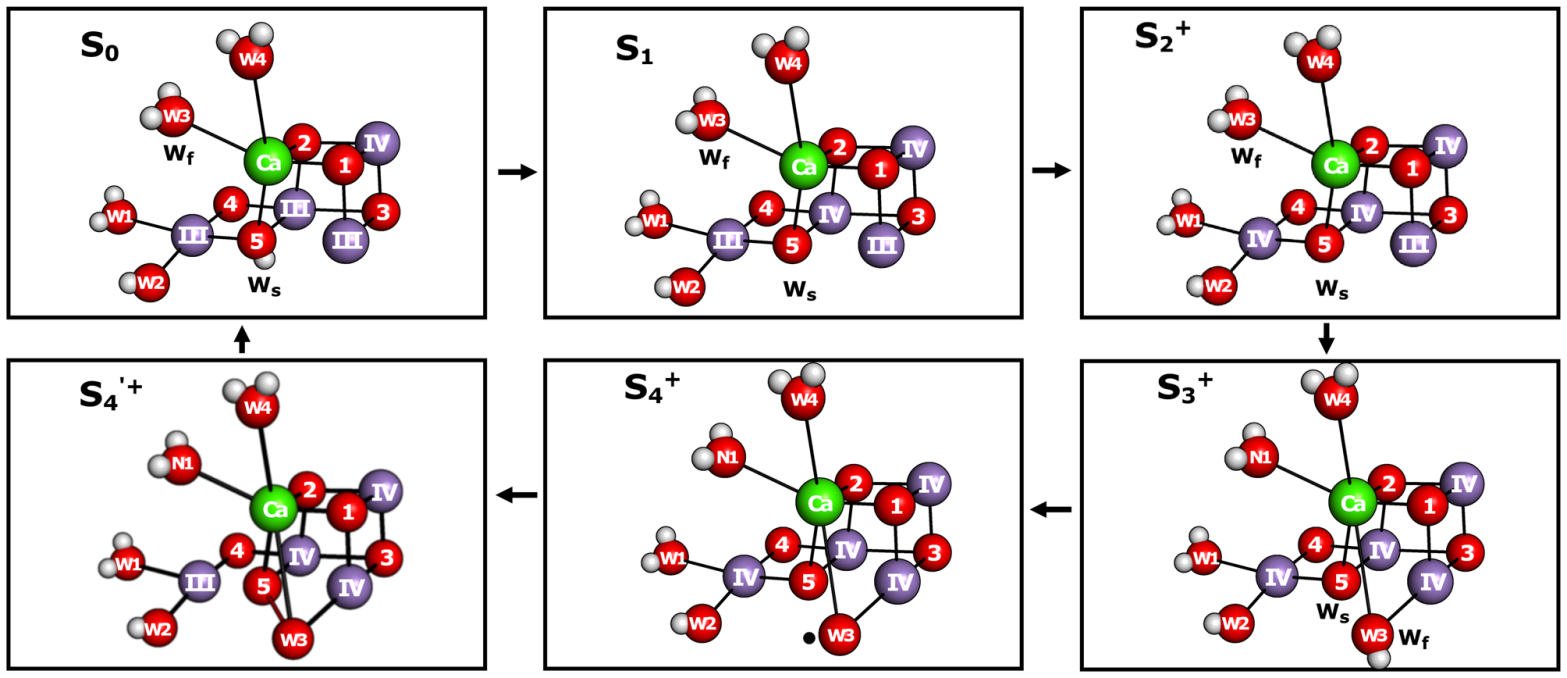
A schematic presentation of the OEC conformations of the S states during the Kok cycle, based on previous spectroscopic, structural, and DFT results, see Text. The oxygens proposed to be the fast and slowly exchanging substrate waters are denoted as W_f_ and W_s_, respectively, for states their exchange rates have been measured. N1 signifies a new water molecule that replaces W3 at Ca during the S_2_ → S_3_ transition. A second water binding event during the reconstruction of the cluster after O_2_ release in the S_4_ → S_0_ transition, as well as proton and O_2_ release are not indicated for simplicity of presentation. Manganese atoms are shown in magenta, calcium in green, and oxygen in red with Arabic numbers. Roman numbers indicate the oxidation states of manganese ions

The analysis obtained here is fully consistent with the picture derived by us over the years by combining substrate-water exchange results with structural information from advanced EPR and snapshot crystallography at XFELs (Messinger 2004; Rapatskiy et al. 2012; Cox and Messinger 2013; Navarro et al. 2013; Nilsson et al. 2014b; Kern et al. 2018; de Lichtenberg and Messinger 2020; Ibrahim et al. 2020; de Lichtenberg et al. 2021; Hussein et al. 2021; Bhowmick et al. 2023). In this model, for substrate-water binding and O–O bond formation (Fig. 4), O5 is the slowly exchanging substrate in all S states, while W3 bound as terminal ligand to Ca may be identified as the fast-exchanging substrate in the S_0_, S_1_, and S_2_ states [although in rapid exchange equilibrium with all other water molecules in the inner cavity (de Lichtenberg et al. 2021)]. In these early S states, W_f_ exchange is limited by isotopic equilibration through barriers in the channels connecting the bulk and the Mn_4_CaO_5_ cluster (Vassiliev et al. 2012; de Lichtenberg et al. 2021), while during the S_2_ → S_3_ transition W_f_ moves into the O_X_/O6 position that bridges Ca and Mn1. The binding of W_f_ to Mn in the S_3_ state is consistent with its slower exchange in S_3_ compared to the earlier S states. Since in the major S_3_ state conformation all Mn ions of the cluster are in the Mn(IV) state (Haumann et al. 2005; Cox et al. 2014; Kern et al. 2018; Schuth et al. 2018) and thus are exchange inert, its rate of exchange may be limited by the back donation of one electron from Y_Z_ (Siegbahn 2013; Nilsson et al. 2014b; de Lichtenberg et al. 2021), i.e., by the formation of the S_2_′Y_Z_^ox^ state. Nevertheless, also S_3_ minority species are proposed in which Mn(III) ions may be formed by partial oxygen ligand oxidation, such as oxyl radical or peroxide formation (Isobe et al. 2019; Corry and O’Malley 2021). While peroxide formation between the substrates is expected to fully block exchange, oxyl radical formation may initiate water exchange under certain conditions.

In principle, also two kinetic phases for W_f_ exchange may be expected and are indeed included in Scheme 1. However, the apparent rates, ${k}_{{\text{f}}1}^{*}$ and ${k}_{{\text{f}}2}^{*}$, are within a factor of 2 (Table 1), consistent with the experimental observation that under most circumstances only one fast phase can be observed. The inability to resolve two fast rates is consistent with the above picture that W_f_ exchange in the S_2_ state is limited by equilibration of all ‘cavity’ waters around the Mn_4_CaO_5_ cluster with bulk water (de Lichtenberg et al. 2021), which effectively prevents the observation of possible chemical differences in W_f_ binding between E_LS_ and E_HS_.

In our model, exchange of W_s_ (O5) in S_3_ state is dependent on Mn(III) formation, e.g., via Y_Z_ back donation, but slower than W_f_ exchange because additional significant barriers are involved for its exchange.

## Conclusion

The present extension of the analytical analysis of the two-state two-conformation model for the S_2_ state (Scheme 1) and its interpretation in the context of literature data reconciles the expected non-exchangeability of O5 when bound between Mn(IV) ions (Tagore et al. 2006, 2007) with the experimental and theoretical assignment of O5 as the slowly exchanging substrate water in all S states (Messinger 2004; Siegbahn 2009; Rapatskiy et al. 2012; Cox and Messinger 2013), and excludes the alternative substrate water assignments in the literature (Vinyard et al. 2013, 2015; Vinyard and Brudvig 2017).

## Supplementary Information

Below is the link to the electronic supplementary material.

Supplementary file1 (DOCX 139 KB)

## Data Availability

Original data will be provided upon request.

## Abbreviations

EPR: Electron paramagnetic resonance
DFT: Density functional theory
E: Conformational state of enzyme
*k*_c_: Rate of equilibration between HS and LS state
*k*_f_, *k*_S_: Fast and slow rate for substrate-water exchange
*k**: Apparent rate constant
HS, LS: High spin, low spin
MIMS: Membrane inlet mass spectrometry
Mn_4_CaO_5/6_: Inorganic cofactor of the OEC with 5 (S_0_–S_2_) or 6 (S_3_, S_4_) oxo bridges
OEC: Oxygen-evolving complex: functional unit of PSII that catalyzes water oxidation
O5: Central oxygen bridge of the Mn_4_CaO_5/6_ cluster
PSII: Photosystem II
S_*i*_ states: Oxidation states of the Mn_4_CaO_5/6_ cluster (*i* = 0, 1, 2, 3, 4)
W_f_, W_S_: Fast and slowly exchanging substrate water bound to the Mn_4_CaO_5/6_ cluster
W_N_: New water added to the Mn_4_CaO_5_ cluster during the S_2_ → S_3_ transition
W1–W4: Terminal water ligands of the OEC
^34^Y, ^36^Y: Oxygen yield at mass-to-charge ratio 34 and 36
XFEL: X-ray free electron laser
*α*_in_, *α*_f_: Initial and final ^18^O isotope enrichment of the buffer

## References

[CR1] Bhowmick A, Hussein R, Bogacz I, Simon PS, Ibrahim M, Chatterjee R, Doyle MD, Cheah MH, Fransson T, Chernev P, Kim I-S, Makita H, Dasgupta M, Kaminsky CJ, Zhang M, Gätcke J, Haupt S, Nangca II, Keable SM, Aydin AO, Tono K, Owada S, Gee LB, Fuller FD, Batyuk A, Alonso-Mori R, Holton JM, Paley DW, Moriarty NW, Mamedov F, Adams PD, Brewster AS, Dobbek H, Sauter NK, Bergmann U, Zouni A, Messinger J, Kern J, Yano J, Yachandra VK (2023) Structural evidence for intermediates during O_2_ formation in photosystem II. Nature 617:629–636. 10.1038/s41586-023-06038-z 37138085 10.1038/s41586-023-06038-z PMC10191843

[CR2] Boussac A, Ugur I, Marion A, Sugiura M, Kaila VRI, Rutherford AW (2018) The low spin–high spin equilibrium in the S_2_-state of the water oxidizing enzyme. Biochim Biophys Acta 1859(5):342–356. 10.1016/j.bbabio.2018.02.010 10.1016/j.bbabio.2018.02.010 29499187

[CR3] Bovi D, Narzi D, Guidoni L (2013) The S_2_ state of the oxygen-evolving complex of photosystem II explored by QM/MM dynamics: spin surfaces and metastable states suggest a reaction path towards the S_3_ state. Angew Chem Int Ed 52(45):11744–11749. 10.1002/anie.201306667 10.1002/anie.201306667 PMC3952239 24115467

[CR4] Chatterjee R, Lassalle L, Gul S, Fuller FD, Young ID, Ibrahim M, de Lichtenberg C, Cheah MH, Zouni A, Messinger J, Yachandra VK, Kern J, Yano J (2019) Structural isomers of the S_2_ state in photosystem II: do they exist at room temperature and are they important for function? Physiol Plant 166(1):60–72. 10.1111/ppl.12947 30793319 10.1111/ppl.12947 PMC6478542

[CR5] Cheah MH, Zhang M, Shevela D, Mamedov F, Zouni A, Messinger J (2020) Assessment of the manganese cluster’s oxidation state via photoactivation of photosystem II microcrystals. Proc Natl Acad Sci USA 117(1):141–145. 10.1073/pnas.1915879117 31848244 10.1073/pnas.1915879117 PMC6955365

[CR6] Corry TA, O’Malley PJ (2019) Proton isomers rationalize the high- and low-spin forms of the S_2_ state intermediate in the water-oxidizing reaction of photosystem II. J Phys Chem Lett 10(17):5226–5230. 10.1021/acs.jpclett.9b01372 31429574 10.1021/acs.jpclett.9b01372

[CR7] Corry TA, O’Malley PJ (2021) S_3_ state models of nature’s water oxidizing complex: analysis of bonding and magnetic exchange pathways, assessment of experimental electron paramagnetic resonance data, and implications for the water oxidation mechanism. J Phys Chem B 125(36):10097–10107. 10.1021/acs.jpcb.1c04459 34463499 10.1021/acs.jpcb.1c04459

[CR8] Cox N, Messinger J (2013) Reflections on substrate water and dioxygen formation. Biochim Biophys Acta 1827:1020–1030. 10.1016/j.bbabio.2013.01.013 23380392 10.1016/j.bbabio.2013.01.013

[CR9] Cox N, Retegan M, Neese F, Pantazis DA, Boussac A, Lubitz W (2014) Electronic structure of the oxygen-evolving complex in photosystem II prior to O-O bond formation. Science 345(6198):804–808. 10.1126/science.1254910 25124437 10.1126/science.1254910

[CR10] Dau H, Limberg C, Reier T, Risch M, Roggan S, Strasser P (2010) The mechanism of water oxidation: from electrolysis via homogeneous to biological catalysis. ChemCatChem 2(7):724–761. 10.1002/cctc.201000126

[CR11] de Lichtenberg C, Messinger J (2020) Substrate water exchange in the S_2_ state of photosystem II is dependent on the conformation of the Mn_4_Ca cluster. Phys Chem Chem Phys 22(23):12894–12908. 10.1039/D0CP01380C 32373850 10.1039/d0cp01380c

[CR12] de Lichtenberg C, Kim CJ, Chernev P, Debus RJ, Messinger J (2021) The exchange of the fast substrate water in the S_2_ state of photosystem II is limited by diffusion of bulk water through channels—implications for the water oxidation mechanism. Chem Sci 12(38):12763–12775. 10.1039/d1sc02265b 34703563 10.1039/d1sc02265b PMC8494045

[CR13] Dismukes GC, Siderer Y (1981) Intermediates of a polynuclear manganese cluster involved in photosynthetic oxidation of water. Proc Natl Acad Sci USA 78(1):274–278. 10.1073/pnas.78.1.274 16592949 10.1073/pnas.78.1.274 PMC319035

[CR14] Drosou M, Zahariou G, Pantazis DA (2021) Orientational Jahn-Teller isomerism in the dark-stable state of Nature’s water oxidase. Angew Chem Int Ed 60(24):13493–13499. 10.1002/anie.202103425 10.1002/anie.202103425 PMC8252073 33830630

[CR15] Greife P, Schönborn M, Capone M, Assunção R, Narzi D, Guidoni L, Dau H (2023) The electron–proton bottleneck of photosynthetic oxygen evolution. Nature 617:623–628. 10.1038/s41586-023-06008-5 37138082 10.1038/s41586-023-06008-5 PMC10191853

[CR16] Haumann M, Müller C, Liebisch P, Iuzzolino L, Dittmer J, Grabolle M, Neisius T, Meyer-Klaucke W, Dau H (2005) Structural and oxidation state changes of the photosystem II manganese complex in four transitions of the water oxidation cycle (S_0_ → S_1_, S_1_ → S_2_, S_2_ → S_3_, and S_3_, S_4_ → S_0_) characterized by X-ray absorption spectroscopy at 20 K and room temperature. Biochemistry 44(6):1894–1908. 10.1021/bi048697e 15697215 10.1021/bi048697e

[CR17] Helm L, Merbach AE (2005) Inorganic and bioinorganic solvent exchange mechanisms. Chem Rev 105(6):1923–1959. 10.1021/cr030726o 15941206 10.1021/cr030726o

[CR18] Hillier W, Wydrzynski T (2000) The affinities for the two substrate water binding sites in the O_2_ evolving complex of photosystem II vary independently during S-state turnover. Biochemistry 39(15):4399–4405. 10.1021/bi992318d 10757989 10.1021/bi992318d

[CR19] Hillier W, Wydrzynski T (2001) Oxygen ligand exchange at metal sites: implications for the O_2_ evolving mechanism of photosystem II. Biochim Biophys Acta 1503(1–2):197–209. 10.1016/S0005-2728(00)00225-5 11115634 10.1016/s0005-2728(00)00225-5

[CR20] Hillier W, Wydrzynski T (2008) ^18^O-Water exchange in photosystem II: substrate binding and intermediates of the water splitting cycle. Coord Chem Rev 252:306–317. 10.1016/j.ccr.2007.09.004

[CR21] Hillier W, Messinger J, Wydrzynski T (1998) Kinetic determination of the fast exchanging substrate water molecule in the S_3_ state of photosystem II. Biochemistry 37(48):16908–16914. 10.1021/bi980756z 9836583 10.1021/bi980756z

[CR22] Huang H-L, Brudvig GW (2021) Kinetic modeling of substrate-water exchange in photosystem II. BBA Adv 1:100014. 10.1016/j.bbadva.2021.100014 37082013 10.1016/j.bbadva.2021.100014 PMC10074954

[CR23] Hussein R, Ibrahim M, Bhowmick A, Simon PS, Chatterjee R, Lassalle L, Doyle M, Bogacz I, Kim IS, Cheah MH, Gul S, de Lichtenberg C, Chernev P, Pham CC, Young ID, Carbajo S, Fuller FD, Alonso-Mori R, Batyuk A, Sutherlin KD, Brewster AS, Bolotovsky R, Mendez D, Holton JM, Moriarty NW, Adams PD, Bergmann U, Sauter NK, Dobbek H, Messinger J, Zouni A, Kern J, Yachandra VK, Yano J (2021) Structural dynamics in the water and proton channels of photosystem II during the S_2_ to S_3_ transition. Nat Commun. 10.1038/s41467-021-26781-z 34764256 10.1038/s41467-021-26781-z PMC8585918

[CR24] Ibrahim M, Fransson T, Chatterjee R, Cheah MH, Hussein R, Lassalle L, Sutherlin KD, Young ID, Fuller FD, Gul S, Kim IS, Simon PS, de Lichtenberg C, Chernev P, Bogacz I, Pham CC, Orville AM, Saichek N, Northen T, Batyuk A, Carbajo S, Alonso-Mori R, Tono K, Owada S, Bhowmick A, Bolotovsky R, Mendez D, Moriarty NW, Holton JM, Dobbek H, Brewster AS, Adams PD, Sauter NK, Bergmann U, Zouni A, Messinger J, Kern J, Yachandra VK, Yano J (2020) Untangling the sequence of events during the S_2_ → S_3_ transition in photosystem II and implications for the water oxidation mechanism. Proc Natl Acad Sci USA 117(23):12624–12635. 10.1073/pnas.2000529117 32434915 10.1073/pnas.2000529117 PMC7293653

[CR25] Isobe H, Shoji M, Yamanaka S, Umena Y, Kawakami K, Kamiya N, Shen JR, Yamaguchi K (2012) Theoretical illumination of water-inserted structures of the CaMn_4_O_5_ cluster in the S_2_ and S_3_ states of oxygen-evolving complex of photosystem II: full geometry optimizations by B3LYP hybrid density functional. Dalton Trans 41(44):13727–13740. 10.1039/C2dt31420g 23037319 10.1039/c2dt31420g

[CR26] Isobe H, Shoji M, Suzuki T, Shen JR, Yamaguchi K (2019) Spin, valence, and structural isomerism in the S_3_ state of the oxygen-evolving complex of photosystem II as a manifestation of multimetallic cooperativity. J Chem Theory Comput 15(4):2375–2391. 10.1021/acs.jctc.8b01055 30855953 10.1021/acs.jctc.8b01055

[CR27] Junge W (2019) Oxygenic photosynthesis: history, status and perspective. Q Rev Biophys. 10.1017/S0033583518000112 30670110 10.1017/S0033583518000112

[CR28] Kern J, Chatterjee R, Young ID, Fuller FD, Lassalle L, Ibrahim M, Gul S, Fransson T, Brewster AS, Alonso-Mori R, Hussein R, Zhang M, Douthit L, de Lichtenberg C, Cheah MH, Shevela D, Wersig J, Seuffert I, Sokaras D, Pastor E, Weninger C, Kroll T, Sierra RG, Aller P, Butryn A, Orville AM, Liang MN, Batyuk A, Koglin JE, Carbajo S, Boutet S, Moriarty NW, Holton JM, Dobbek H, Adams PD, Bergmann U, Sauter NK, Zouni A, Messinger J, Yano J, Yachandra VK (2018) Structures of the intermediates of Kok’s photosynthetic water oxidation clock. Nature 563(7731):421–425. 10.1038/s41586-018-0681-2 30405241 10.1038/s41586-018-0681-2 PMC6485242

[CR29] Kim DH, Britt RD, Klein MP, Sauer K (1992) The manganese site of the photosynthetic oxygen-evolving complex probed by EPR spectroscopy of oriented photosystem II membranes: the *g* = 4 and *g* = 2 multiline signals. Biochemistry 31:541–547. 10.1021/bi00117a034 1310041 10.1021/bi00117a034

[CR30] Kok B, Forbush B, McGloin M (1970) Cooperation of charges in photosynthetic O_2_ evolution. Photochem Photobiol 11:457–476. 10.1111/j.1751-1097.1970.tb06017.x 5456273 10.1111/j.1751-1097.1970.tb06017.x

[CR31] Krewald V, Retegan M, Cox N, Messinger J, Lubitz W, DeBeer S, Neese F, Pantazis DA (2015) Metal oxidation states in biological water splitting. Chem Sci 6(3):1676–1695. 10.1039/c4sc03720k 29308133 10.1039/c4sc03720k PMC5639794

[CR32] Kulik LV, Epel B, Lubitz W, Messinger J (2007) Electronic structure of the Mn_4_O_x_Ca cluster in the S_0_ and S_2_ states of the oxygen-evolving complex of photosystem II based on pulse ^55^Mn-ENDOR and EPR spectroscopy. J Am Chem Soc 129:13421–13435. 10.1021/ja071487f 17927172 10.1021/ja071487f

[CR33] Li XC, Siegbahn PEM (2015) Alternative mechanisms for O_2_ release and O-O bond formation in the oxygen evolving complex of photosystem II. Phys Chem Chem Phys 17(18):12168–12174. 10.1039/c5cp00138b 25879997 10.1039/c5cp00138b

[CR34] Li HJ, Nakajima Y, Nomura T, Sugahara M, Yonekura S, Chan SK, Nakane T, Yamane T, Umena Y, Suzuki M, Masuda T, Motomura T, Naitow H, Matsuura Y, Kimura T, Tono K, Owada S, Joti Y, Tanaka R, Nango E, Akita F, Kubo M, Iwata S, Shen JR, Suga M (2021) Capturing structural changes of the S_1_ to S_2_ transition of photosystem II using time-resolved serial femtosecond crystallography. IUCrJ 8:431–443. 10.1107/S2052252521002177 33953929 10.1107/S2052252521002177 PMC8086164

[CR35] Lubitz W, Chrysina M, Cox N (2019) Water oxidation in photosystem II. Photosynth Res 142(1):105–125. 10.1007/s11120-019-00648-3 31187340 10.1007/s11120-019-00648-3 PMC6763417

[CR36] Messinger J (2004) Evaluation of different mechanistic proposals for water oxidation in photosynthesis on the basis of Mn_4_O_x_Ca structures for the catalytic site and spectroscopic data. Phys Chem Chem Phys 6:4764–4771. 10.1039/B406437B

[CR37] Messinger J, Badger M, Wydrzynski T (1995) Detection of *one* slowly exchanging substrate water molecule in the S_3_ state of photosystem II. Proc Natl Acad Sci USA 92:3209–3213. 10.1073/pnas.92.8.3209 11607525 10.1073/pnas.92.8.3209 PMC42135

[CR38] Navarro MP, Ames WM, Nilsson H, Lohmiller T, Pantazis DA, Rapatskiy L, Nowaczyk MM, Neese F, Boussac A, Messinger J, Lubitz W, Cox N (2013) Ammonia binding to the oxygen-evolving complex of photosystem II identifies the solvent-exchangeable oxygen bridge (μ-oxo) of the manganese tetramer. Proc Natl Acad Sci USA 110(39):15561–15566. 10.1073/pnas.1304334110 24023065 10.1073/pnas.1304334110 PMC3785721

[CR39] Nilsson H, Krupnik T, Kargul J, Messinger J (2014a) Substrate water exchange in photosystem II core complexes of the extremophilic red alga *Cyanidioschyzon merolae*. Biochim Biophys Acta 1837(8):1257–1262. 10.1016/j.bbabio.2014.04.001 24726350 10.1016/j.bbabio.2014.04.001

[CR40] Nilsson H, Rappaport F, Boussac A, Messinger J (2014b) Substrate-water exchange in photosystem II is arrested before dioxygen formation. Nat Commun 5:4305. 10.1038/ncomms5305 24993602 10.1038/ncomms5305 PMC4102119

[CR41] Pantazis DA (2018) Missing pieces in the puzzle of biological water oxidation. ACS Catal 8(10):9477–9507. 10.1021/acscatal.8b01928

[CR42] Pantazis DA, Ames W, Cox N, Lubitz W, Neese F (2012) Two interconvertible structures that explain the spectroscopic properties of the oxygen-evolving complex of photosystem II in the S_2_ state. Angew Chem Int Ed 51(39):9935–9940. 10.1002/anie.201204705 10.1002/anie.201204705 22907906

[CR43] Pushkar Y, Ravari AK, Jensen SC, Palenik M (2019) Early binding of substrate oxygen is responsible for a spectroscopically distinct S_2_ state in photosystem II. J Phys Chem Lett 10(17):5284–5291. 10.1021/acs.jpclett.9b01255 31419136 10.1021/acs.jpclett.9b01255

[CR44] Rapatskiy L, Cox N, Savitsky A, Ames WM, Sander J, Nowaczyk MM, Rögner M, Boussac A, Neese F, Messinger J, Lubitz W (2012) Detection of the water-binding sites of the oxygen-evolving complex of photosystem II using W-band ^17^O electron-electron double resonance-detected NMR spectroscopy. J Am Chem Soc 134(40):16619–16634. 10.1021/Ja3053267 22937979 10.1021/ja3053267

[CR45] Schuth N, Zaharieva I, Chernev P, Berggren G, Anderlund M, Styring S, Dau H, Haumann M (2018) K alpha X-ray emission spectroscopy on the photosynthetic oxygen-evolving complex supports manganese oxidation and water binding in the S_3_ state. Inorg Chem 57(16):10424–10430. 10.1021/acs.inorgchem.8b01674 10.1021/acs.inorgchem.8b01674 30067343

[CR46] Shevela D, Kern JF, Govindjee G, Messinger J (2023) Solar energy conversion by photosystem II: principles and structures. Photosynth Res 156(3):279–307. 10.1007/s11120-022-00991-y 36826741 10.1007/s11120-022-00991-y PMC10203033

[CR47] Siegbahn PEM (2009) Structures and energetics for O_2_ formation in photosystem II. Acc Chem Res 42(12):1871–1880. 10.1021/Ar900117k 19856959 10.1021/ar900117k

[CR48] Siegbahn PEM (2013) Substrate water exchange for the oxygen evolving complex in PSII in the S_1_, S_2_, and S_3_ states. J Am Chem Soc 135(25):9442–9449. 10.1021/ja401517e 23742698 10.1021/ja401517e

[CR49] Suga M, Akita F, Hirata K, Ueno G, Murakami H, Nakajima Y, Shimizu T, Yamashita K, Yamamoto M, Ago H, Shen JR (2015) Native structure of photosystem II at 1.95 Å resolution viewed by femtosecond x-ray pulses. Nature 517(7532):99–103. 10.1038/nature13991 25470056 10.1038/nature13991

[CR50] Suga M, Akita F, Sugahara M, Kubo M, Nakajima Y, Nakane T, Yamashita K, Umena Y, Nakabayashi M, Yamane T, Nakano T, Suzuki M, Masuda T, Inoue S, Kimura T, Nomura T, Yonekura S, Yu LJ, Sakamoto T, Motomura T, Chen JH, Kato Y, Noguchi T, Tono K, Joti Y, Kameshima T, Hatsui T, Nango E, Tanaka R, Naitow H, Matsuura Y, Yamashita A, Yamamoto M, Nureki O, Yabashi M, Ishikawa T, Iwata S, Shen JR (2017) Light-induced structural changes and the site of O=O bond formation in PSII caught by XFEL. Nature 543(7643):131–135. 10.1038/nature21400 28219079 10.1038/nature21400

[CR51] Suga M, Akita F, Yamashita K, Nakajima Y, Ueno G, Li HJ, Yamane T, Hirata K, Umena Y, Yonekura S, Yu LJ, Murakami H, Nomura T, Kimura T, Kubo M, Baba S, Kumasaka T, Tono K, Yabashi M, Isobe H, Yamaguchi K, Yamamoto M, Ago H, Shen JR (2019) An oxyl/oxo mechanism for oxygen-oxygen coupling in PSII revealed by an x-ray free-electron laser. Science 366(6463):334–338. 10.1126/science.aax6998 31624207 10.1126/science.aax6998

[CR52] Tagore R, Chen HY, Crabtree RH, Brudvig GW (2006) Determination of μ-oxo exchange rates in di-μ-oxo dimanganese complexes by electrospray ionization mass spectrometry. J Am Chem Soc 128(29):9457–9465. 10.1021/ja061348i 16848483 10.1021/ja061348i

[CR53] Tagore R, Crabtree RH, Brudvig GW (2007) Distinct mechanisms of bridging-oxo exchange in di-μ-O dimanganese complexes with and without water-binding sites: implications for water binding in the O_2_-evolving complex of photosystem II. Inorg Chem 46(6):2193–2203. 10.1021/ic061968k 17295472 10.1021/ic061968k

[CR54] Tanaka A, Fukushima Y, Kamiya N (2017) Two different structures of the oxygen-evolving complex in the same polypeptide frameworks of photosystem II. J Am Chem Soc 139(5):1718–1721. 10.1021/jacs.6b09666 28102667 10.1021/jacs.6b09666

[CR55] Umena Y, Kawakami K, Shen JR, Kamiya N (2011) Crystal structure of oxygen-evolving photosystem II at a resolution of 1.9 Å. Nature 473(7345):55–61. 10.1038/Nature09913 21499260 10.1038/nature09913

[CR56] Vassiliev S, Zaraiskaya T, Bruce D (2012) Exploring the energetics of water permeation in photosystem II by multiple steered molecular dynamics simulations. Biochim Biophys Acta 1817(9):1671–1678. 10.1016/j.bbabio.2012.05.016 22683291 10.1016/j.bbabio.2012.05.016

[CR57] Vinyard DJ, Brudvig GW (2017) Progress toward a molecular mechanism of water oxidation in photosystem II. Annu Rev Phys Chem 68(1):101–116. 10.1146/annurev-physchem-052516-044820 28226223 10.1146/annurev-physchem-052516-044820

[CR58] Vinyard DJ, Ananyev GM, Dismukes GC (2013) Photosystem II: the reaction center of oxygenic photosynthesis. Annu Rev Biochem 82:577–606. 10.1146/annurev-biochem-070511-100425 23527694 10.1146/annurev-biochem-070511-100425

[CR59] Vinyard DJ, Khan S, Brudvig GW (2015) Photosynthetic water oxidation: binding and activation of substrate waters for O-O bond formation. Faraday Discuss 185:37–50. 10.1039/c5fd00087d 26447686 10.1039/c5fd00087d

[CR60] Vinyard DJ, Khan S, Askerka M, Batista VS, Brudvig GW (2017) Energetics of the S_2_ state spin isomers of the oxygen-evolving complex of photosystem II. J Phys Chem B 121(5):1020–1025. 10.1021/acs.jpcb.7b00110 28079373 10.1021/acs.jpcb.7b00110

[CR61] Wang J, Armstrong WH, Batista VS (2021) Do crystallographic XFEL data support binding of a water molecule to the oxygen-evolving complex of photosystem II exposed to two flashes of light? Proc Natl Acad Sci USA 118(24):e2023982118. 10.1073/pnas.2023982118 34117119 10.1073/pnas.2023982118 PMC8214663

[CR62] Yachandra VK, Sauer K, Klein MP (1996) Manganese cluster in photosynthesis: where plants oxidize water to dioxygen. Chem Rev 96:2927–2950. 10.1021/cr950052k 11848846 10.1021/cr950052k

[CR63] Yamaguchi K, Miyagawa K, Shoji M, Isobe H, Kawakami T (2022a) Elucidation of a multiple S_3_ intermediates model for water oxidation in the oxygen evolving complex of photosystem II. Calcium-assisted concerted O-O bond formation. Chem Phys Lett. 10.1016/j.cplett.2022.140042

[CR64] Yamaguchi K, Shoji M, Isobe H, Kawakami T, Miyagawa K, Suga M, Akita F, Shen JR (2022b) Geometric, electronic and spin structures of the CaMn_4_O_5_ catalyst for water oxidation in oxygen-evolving photosystem II. Interplay between experiments and theoretical computations. Coord Chem Rev. 10.1016/j.ccr.2022.214742

[CR65] Young ID, Ibrahim M, Chatterjee R, Gul S, Fuller FD, Koroidov S, Brewster AS, Tran R, Alonso-Mori R, Kroll T, Michels-Clark T, Laksmono H, Sierra RG, Stan CA, Hussein R, Zhang M, Douthit L, Kubin M, de Lichtenberg C, Pham LV, Nilsson H, Cheah MH, Shevela D, Saracini C, Bean MA, Seuffert I, Sokaras D, Weng TC, Pastor E, Weninger C, Fransson T, Lassalle L, Brauer P, Aller P, Docker PT, Andi B, Orville AM, Glownia JM, Nelson S, Sikorski M, Zhu DL, Hunter MS, Lane TJ, Aquila A, Koglin JE, Robinson J, Liang MN, Boutet S, Lyubimov AY, Uervirojnangkoorn M, Moriarty NW, Liebschner D, Afonine PV, Waterman DG, Evans G, Wernet P, Dobbek H, Weis WI, Brunger AT, Zwart PH, Adams PD, Zouni A, Messinger J, Bergmann U, Sauter NK, Kern J, Yachandra VK, Yano J (2016) Structure of photosystem II and substrate binding at room temperature. Nature 540(7633):453–457. 10.1038/nature20161 27871088 10.1038/nature20161 PMC5201176

[CR66] Zimmermann JL, Rutherford AW (1984) EPR studies of the oxygen-evolving enzyme of photosystem II. Biochim Biophys Acta 767(1):160–167. 10.1016/0005-2728(84)90091-4

