# Supplementary material for: On the simulation and interpretation of substrate-water exchange experiments in photosynthetic water oxidation
Source: Photosynth Res. 2024 Mar 21;162(2-3):413–26. doi: 10.1007/s11120-024-01084-8 (PMC11639282; doi:10.1007/s11120-024-01084-8)
Supplement: Supplementary file 1 — Supplementary file1 (DOCX 139 KB) [file 11120_2024_1084_MOESM1_ESM.docx]

**On the simulation and interpretation of substrate water exchange experiments in photosynthetic water oxidation**

Petko Chernev, A. Orkun Aydin, Johannes Messinger*

Molecular Biomimetics, Department of Chemistry – Ångström Laboratory, 75120 Uppsala, Sweden

*Corresponding author: johannes.messinger@kemi.uu.se

**Supporting information**

The equations for the exponential terms in the model of Huang and Brudvig (Huang and Brudvig 2021), are given by

$${}^{34}{Y\propto\left[ E_{LS}^{16,18} \right]+\left[ E_{LS}^{18,16} \right]+\left[ E_{HS}^{16,18} \right]+\left[ E_{HS}^{18,16} \right]=\frac{2r}{\left( 1+r \right)^{2}}\left[ -\frac{1}{r}C_{1}\left( t \right)-\frac{r-1}{2r}C_{2}\left( t \right)-\frac{r-1}{2r}C_{3}\left( t \right)+1 \right]}$$

$${}^{36}{Y\propto\left[ E_{LS}^{18,18} \right]+\left[ E_{HS}^{18,18} \right]=\frac{1}{\left( 1+r \right)^{2}}\left[ C_{1}\left( t \right)-C_{2}\left( t \right)-C_{3}\left( t \right)+1 \right]}$$

$$C_{i}(t)=c_{i}^{+}e^{\lambda_{i}^{+}t}+c_{i}^{-}e^{\lambda_{i}^{-}t}$$

$$c_{i}^{+,-}=\frac{1}{2}\pm\frac{1}{2\left( \lambda_{i}^{+}-\lambda_{i}^{-} \right)}\left[ k_{c1}+k_{c2}+\frac{\left( k_{c1}-k_{c2} \right)\left[ x\left( -k_{f1}^{*}+k_{f2}^{*} \right)+y\left( {-k}_{s1}^{*}+k_{s2}^{*} \right) \right]}{k_{c1}+k_{c2}} \right]$$

$$\left[ \begin{aligned} x=1, y=1 \mathrm{if} i=1 \\ x=1, y=0 \mathrm{if} i=2 \\ x=0, y=1 \mathrm{if} i=3 \end{aligned} \right.$$

with the *λ*’s in the exponential terms being the eigenvalues of the kinetic rate constant matrix describing the model:

$$\lambda_{1}^{+,-}=\frac{1}{2}\left[ -\left( k_{f1}^{*}+k_{s1}^{*}+k_{f2}^{*}+k_{s2}^{*}+k_{c1}+k_{c2} \right)\pm\sqrt{\left( k_{f1}^{*}+k_{s1}^{*}+k_{f2}^{*}+k_{s2}^{*}+k_{c1}+k_{c2} \right)^{2}-4\left[ \left( k_{f1}^{*}+k_{s1}^{*} \right)\left( k_{f2}^{*}+k_{s2}^{*} \right)+\left( k_{f1}^{*}+k_{s1}^{*} \right)k_{c1}+\left( k_{f2}^{*}+k_{s2}^{*} \right)k_{c2} \right]} \right]$$

$$\lambda_{2}^{+,-}=\frac{1}{2}\left[ -\left( k_{f1}^{*}+k_{f2}^{*}+k_{c1}+k_{c2} \right)\pm\sqrt{\left( k_{f1}^{*}+k_{f2}^{*}+k_{c1}+k_{c2} \right)^{2}-4\left[ \left( k_{f1}^{*}k_{f2}^{*}+k_{f1}^{*}k_{c1}+k_{f2}^{*}k_{c2} \right) \right]} \right]$$

$$\lambda_{3}^{+,-}=\frac{1}{2}\left[ -\left( k_{s1}^{*}+k_{s2}^{*}+k_{c1}+k_{c2} \right)\pm\sqrt{\left( k_{s1}^{*}+k_{s2}^{*}+k_{c1}+k_{c2} \right)^{2}-4\left[ \left( k_{s1}^{*}k_{s2}^{*}+k_{s1}^{*}k_{c1}+k_{s2}^{*}k_{c2} \right) \right]} \right]$$

$$\lambda_{4}=-\left( k_{c1}+k_{c2} \right)$$

$$\lambda_{5}=0$$

The first three pairs of eigenvalues can also be rewritten as

$$\lambda_{1}^{+,-}=\frac{1}{2}\left[ -\left( k_{f1}^{*}+k_{s1}^{*}+k_{f2}^{*}+k_{s2}^{*}+k_{c1}+k_{c2} \right)\pm\sqrt{\left( k_{f1}^{*}+k_{s1}^{*}-k_{f2}^{*}-k_{s2}^{*}-k_{c1}+k_{c2} \right)^{2}+4k_{c1}k_{c2}} \right]$$

$$\lambda_{2}^{+,-}=\frac{1}{2}\left[ -\left( k_{f1}^{*}+k_{f2}^{*}+k_{c1}+k_{c2} \right)\pm\sqrt{\left( k_{f1}^{*}-k_{f2}^{*}-k_{c1}+k_{c2} \right)^{2}+4k_{c1}k_{c2}} \right]$$

$\lambda_{3}^{+,-}=\frac{1}{2}\left[ -\left( k_{s1}^{*}+k_{s2}^{*}+k_{c1}+k_{c2} \right)\pm\sqrt{\left( {-k}_{s1}^{*}+k_{s2}^{*}+k_{c1}-k_{c2} \right)^{2}+4k_{c1}k_{c2}} \right]$

d

c

a

b

**Figure S1**. Substrate-water exchange data in Ca^2+^ PSII at pH 8.6 in the S_2_ state taken from (de Lichtenberg and Messinger 2020) and simulated using our extended version of the double-conformation model by (Huang and Brudvig 2021). The single-^18^O-labeled O_2_ yield (m/z = 34) is shown on the left, and the double-^18^O-labeled O_2_ yield (m/z = 36) is shown on the right. Black dots show the experimental data. Black curves show the simulation using the original double-conformation model [Huang 2021] with rate constants $k_{f1}^{*}=94,k_{s1}^{*}=1.1, k_{f2}^{*}=73, k_{s2}^{*}=11.7, k_{c1}=0.084, k_{c2}=0.62$ s^-1^, without applying any corrections. The blue curves show the same simulation when the corrections for initial enrichment (panel a, $\alpha_{\mathrm{in}}$ = 0.7 %), non-instant injection (panel b, *t_k_* = 3 ms), exchange in S_3_ (panel c, with additional 10 ms exchange using rate constants $k_{f}^{*}=19.5,k_{s}^{*}=0.25$ s^-1^), or all three corrections (d) are applied.

**Figure S2**. The value of the time shift $t_{k}$ for a mixing system with $t_{m}$ = 6 ms (eq 11 in main text) for exchange rates *k* in a relevant range between 0.1 and 400 s^-1^. $t_{k}$ is close to $t_{m}$/2 = 3 ms, but increases for high *k*, with $t_{k}$ = 3.3 ms for *k* = 200 s^-1^, and $t_{k}$ = 3.57 ms for *k* = 400 s^-1^.

**Figure S3**. Substrate-water exchange data in Sr^2+^ PSII at pH 6.0 in the S_2_ state taken from (de Lichtenberg and Messinger 2020) and simulated using our extended version of the double-conformation model by (Huang and Brudvig 2021). Only the double-^18^O-labeled O_2_ yield (m/z = 36) is shown. Black dots show the experimental data. The black and the blue curves show the simulation using rate constants given in Table 1 in the main text, row 3 and 4 correspondingly, for the cases when the slow kinetic component (visible at around 1 s H_2_^18^O incubation time, marked by a gray vertical line) corresponds either to the slow water exchange in conformation 1 (black) or to the interconversion rate constant (blue). The intermediate kinetic component (visible at around 0.04 s H_2_^18^O incubation time, marked by a gray vertical line) is explained in both simulations by the slow water exchange in conformation 2 (E_HS_).

**Figure S4**. Bootstrapping distributions of 5000 fits of the model for Ca PSII at pH 8.6 shown in Figure 3A (blue curve) and Table 1 (row 2).

**Figure S5**. Bootstrapping distributions of 5000 fits of the model for Sr PSII at pH 6.0 shown in Figure 3B (blue curve) and Table 1 (row 4).

**Figure S6**. Bootstrapping distributions of 5000 fits of the model for Sr PSII at pH 8.3 shown in Figure 3C (black curve) and Table 1 (row 5).

**References**

de Lichtenberg C, Messinger J (2020) Substrate water exchange in the S_2_ state of photosystem II is dependent on the conformation of the Mn_4_Ca cluster. Phys Chem Chem Phys 22 (23):12894-12908

Huang H-L, Brudvig GW (2021) Kinetic modeling of substrate-water exchange in Photosystem II. BBA Advances 1:100014. doi:<https://doi.org/10.1016/j.bbadva.2021.100014>
